# Caring too much? Lack of public services to older people reduces attendance at work among their children

**DOI:** 10.1007/s10433-016-0403-2

**Published:** 2016-10-31

**Authors:** Heidi Gautun, Christopher Bratt

**Affiliations:** 1 Norwegian Social Research (NOVA), Oslo and Akershus University College (HiOA), Oslo, Norway; 2 University of Kent, Canterbury, UK

**Keywords:** Care for ageing parents, Home care services, Nursing homes, Labour market participation

## Abstract

The need to provide care for older people can put a strain on their adult children, potentially interfering with their work attendance. We tested the hypothesis that public care for older people (nursing homes or home care services) would moderate the association between having an older parent in need of care and reduced work attendance among the adult children. The analysis used data from a survey of Norwegian employees aged 45–65 (*N* = 529). Institutional care for older people in need of care (i.e. nursing homes) was associated with improved work attendance among their children—their daughters in particular. Data also indicated a moderating effect: the link between the parents’ reduced health and reduced work attendance among the children was weaker if the parent lived in a nursing home. However, the results were very different for home-based care: data indicated no positive effects on adult children’s work attendance when parents received non-institutionalised care of this kind. Overall, the results suggest that extending public care service to older people can improve their children’s ability to combine work with care for parents. However, this effect seems to require the high level of care commonly provided by nursing homes. Thus, the current trend towards de-institutionalising care in Europe (and Norway in particular) might hamper work attendance among care-giving adult children, women in particular. Home care services to older people probably need to be extended if they are intended as a real alternative to institutional care.

A number of studies since the early 1970s have shown that public services and the reorganisation of working life can help mothers and fathers combine caring for their infants with participation in employment (Leira 1996). In the 1990s, researchers started to focus on how adult children’s care obligations to elderly parents affect their participation in the labour market. The majority of the research has been undertaken in the USA and UK (Ettner 1996; Evandrou et al. 2002; Finch and Mason 1990; Kröger and Yeandle 2013; Lilly et al. 2007; Pavalko and Artis 1997; Phillips et al. 2002; Wolf and Soldo 1994). However, recent research has been extended to include many other countries (Carmichael and Charles 2003; Gautun and Hagen 2010; Heitmueller and Inglis 2007; Jakobsson et al. 2013; Kalmijn and Saraceno 2008; Kotsadam 2011; Lilly et al. 2007; Mehdizadeh 2015; Naldini et al. 2014; Viitanen 2007). Research shows that the need to care for older parents can result in care-giving adult children either dropping out of the labour market or reducing work participation by cutting down on working hours (Carers UK 2016; Kotsadam 2011; Pavalko and Artis 1997; Phillips 1994; Wolf and Soldo 1994), taking days off (Jenson and Jacobzone 2000) or lessening their focus at work (Carers UK 2016; Jenson and Jacobzone 2000; Pavalko and Henderson 2006; Yeandle et al. 2007).

Knowledge is still scant on which policies facilitate work participation and occupational effort among adults caring for their parents (Kröger and Yeandle 2013). The research literature has referred to four types of political measures that may ease the combination of work on the one hand and caring for elderly parents on the other (Gautun and Hagen 2010; Schmid et al. 2012). These are (1) labour market policies, (2) various forms of financial support schemes for care providers, (3) legal obligations to provide or co-finance care for parents and (4) public care services to older people.

To our knowledge, this article is the first to investigate the connection between older people’s use of public care services and their adult children’s attendance at work. Most studies investigating the connection between informal caring for parents and gainful employment have focused on a potential dropout from the labour market or a tendency to work part time. However, having to care for elderly parents may also reduce adult children’s attendance at work, with possible detrimental effects on overall productivity and the caregiver’s promotion and salary (Bolin et al. 2008; Phillips et al. 2002). We test the hypothesis that the detrimental effect on attendance at work of having an older parent in need of care is moderated (reduced) by the parent’s use of a public nursing homes, possibly also by home care services.

Our data are collected in Norway, a country with traditionally high levels of public care services for older people funded by local authorities (Daatland 2001; Esping-Andersen 2002). Even the very few commercially driven services for older people are funded by local authorities (Blåka et al. 2012; Herning 2012; Vabø et al. 2013). The allocation of home-based services or a stay in a nursing home is decided by a municipality’s health adminstration.

## An increasing elderly population in need for care

Many countries face an ageing population, which represents a demographic challenge (Eurostat 2015; McKnight 2006; Payne et al. 2007; Spillman and Pezzin 2000). An ageing population implies that each person of employable age will have to provide for more old-age pensioners (Bolin et al. 2008; Heitmueller and Inglis 2007; Karoly and Panis 2004). In Norway, extrapolations from current demographic trends indicate that the number of people of working age per person over 67 will be reduced from 4.7 in 2000 to 3.5 in 2030 and 2.9 in 2050 (Norwegian Ministry of Health and Care Services 2006). This imbalance between the older population and people of employable age implies a need to have as many people as possible of employable age working full time until they reach the age of retirement. The increasing number of older people is also expected to increase the demand for health and care services. Even in societies with a developed welfare state, long-term care remains partly a duty of the family; care is to some extent a responsibility shared by family and the welfare state (Daatland 2001; WHO 2002). Due to the population overall getting older, both parties will experience a reduction in the human resources available for caring. With human resources for care becoming a scarce resource split between public and family care, the need for a larger workforce in the public care sector is likely to be accompanied by an expectation that the family will still provide care for its oldest members (Bauer and Sousa-Poza 2015; Gautun and Hagen 2010).

Previous research shows that the children of older family members are important care providers (Gautun and Hagen 2010; Hansen et al. 2013; Kröger and Yeandle 2013; Lilly et al. 2007; Naldini et al. 2014). Older people often need help due to functional impairment (Romøren 2004). Research shows that daughters of older people provide most of the non-professional care, in particular by helping with the most demanding and intensive care (Arber and Ginn 1995; Bauer and Sousa-Poza 2015; Gautun and Hagen 2010; Gautun et al. 2012; Hansen et al. 2013; Kotsadam 2011; Lilly et al. 2007; Schmid et al. 2012). Since women increasingly participate in the labour market, many women in their fifties and sixties are now both working and having to care for their parents (Gautun and Hagen 2010).

## Caring for elderly parents might affect labour market participation

Studies show that giving care to older parents may result in an exit from the labour market, increased use of part-time work or absence from work. This is particularly true for women; female caregivers are more likely than men to withdraw completely from the labour market, and they also work fewer hours if active (Bauer and Sousa-Poza 2015; Jolanki et al. 2013; Kotsadam 2011; Lilly et al. 2007). Although estimates vary, studies agree that some women leave the labour market to help parents or have to quit work because of health problems after prolonged attempts to combine work with care for an older parent (Carmichael and Charles 2003; Jenson and Jacobzone 2000; Kotsadam 2011; Lilly et al. 2007; Phillips 1994; Schmid et al. 2012).

Research also shows that having to care for older parents can hinder children from participating in social arrangements or accepting extra duties that would promote their careers (Gautun and Hagen 2010; Mooney et al. 2002; Phillips 1994). Moreover, employees report that they worry about their parents and may consequently have difficulty concentrating at work (Gautun and Hagen 2010; Phillips et al. 2002). Some caregivers also have to take leave or reduce working hours because they need to follow their older parent to the doctor or to be available during emergencies (Bolin et al. 2008; Phillips et al. 2002).

Analyses of data from Statistics Norway confirm that if the last alive parent is at a terminal stage, his or her child is often absent from work and has reduced income. As a consequence, their children tend to take out much sick leave, and other social security transfers also increase significantly during the terminal stages of their parents’ lives (Fevang et al. 2012).

## Welfare state policies, intergenerational support and female labour market participation

Public expenditures on eldercare appear to affect both intergenerational support and female labour market participation (Crespo 2006; Crespo and Mira 2014; Jenson and Jacobzone 2000; Naldini et al. 2014). The Nordic countries have comprehensive public care services for older people (Esping-Andersen 2002; Kröger and Yeandle 2013; Naldini et al. 2014) and relatively few women withdraw completely from the labour market to care for their older parents (Gautun and Hagen 2010). In contrast, care in southern Europe is predominantly provided by the family, with women assuming the bulk of the responsibility (Crespo and Mira 2014). Countries in Eastern Europe tend to fall somewhere in between in terms of both the level of public expenditure and women’s need to reduce work participation to care for parents (Crespo and Mira 2014). Even here, the family assumes the major responsibility for providing eldercare (Naldini et al. 2014). A third solution can be seen in liberal welfare states such as the United Kingdom. The UK has public care services for older people, but a significant part of the care is provided by a privatised commercial market.

While all the Scandinavian countries are characterised by a generous welfare state and high female labour market participation, there are still differences. Women’s labour market participation is more affected by giving care to parents in Denmark than in Sweden, with Norway in an intermediate position (Jakobsson et al. 2013). These variations have been explained by differences in the organisation of care for older people. Denmark more than the other two countries has focused on home care services for older people (i.e. outside institutions), and Sweden more than the other two countries has focused on giving care for older people within institutions (Jakobsson et al. 2013). Service coverage is lower in Southern and Eastern European countries and intergenerational family care obligations are overall higher, resulting in lower employment among women (Kalmijn and Saraceno 2008).

Research on how public services to older people are related to their children’s labour market participation has mainly analysed data at the national aggregated level. The present research investigates (1) the care children of older people provide to their parents, (2) the parents’ use of public care and (3) the children’s attendance at work. We also extend the research by studying the links between older people’s use of *different* care services and their children’s attendance at work. The need to assist a parent can reduce labour market participation or attendance at work, but this link between having parents in need of care and one’s own attendance at work might depend on whether the parent lives in an institution for older people or receives home care services outside any institution. It will be easier for children to attend work and concentrate at work if they know that their parents are receiving the help they need.

Longitudinal studies in Norway have shown that an increasing number of sons contribute to care for older people (Lingsom 1997; Romøren 2004). Thus, the present research includes both daughters and sons of older people in need of care. We expect to find differences between women and men as employees and caregivers. Daughters tend to provide more care for their older parents and previous research has indicated that women are more likely to drop out of work or reduce attendance at work to be able to care for their parents (Herlofson and Ugreninov 2014). We compare women and men and hypothesise that public care moderates the effects of having a parent in need of care, in particular for women.

## The present research

The current research uses self-reports from children of a parent in need of care. We limit our analysis to those with only one parent alive (a spouse might take responsibility for most of the care if both are alive). We assume that having a parent in need of care is associated with more absence from work. However, we assume a moderating effect by public services and test the following hypothesis:

### **H1**

The association between having a parent in need of care and reduced attendance at work is lower if the parent lives in a nursing home; possibly also if the parent lives in a private home but receives home care services.

Previous research has shown that caring for an elderly parent is associated with women’s reduced participation in the labour market and reduced attendance at work. Thus, we expect the current research to find that the link between having an elderly parent in need of care and work absence is stronger for women than for men. We also hypothesise that:

### **H2**

The moderating effect of public services for older people (moderating the link between having a parent in need of care and work absence) is stronger for women than for men.

## Methods

### Sample

A nationwide sample of 2000 Norwegian men and women aged 45–65, with one or two parents alive, was selected to participate in the survey (with equal distribution between the genders). The sample selection was random and conducted by Statistics Norway (responsible for official statistics in Norway), which also performed the data collection. A total of 944 completed the questionnaire, giving a response rate of 47%. Motivating people to respond to surveys has become more difficult, raising the question of whether the findings can be representative of the population as a whole (Amundsen 2013). Using data from Statistics Norway, we investigated whether responders differed from the general population in terms of gender, education and place of residence. Women and people with higher levels of education were overrepresented in the sample analysed. However, rural communities, towns and cities of various sizes were proportionally represented.

One-third of the respondents had both parents alive at the time of the survey, 57% had only their mothers alive and one in ten had only a living father. We limited the analysis to respondents who were gainfully employed and had one (and only one) parent alive (*N* = 529, aged 45 to 65 years, mean age = 54.3 [*SD* = 4.70], 52% women). People who have parents in need of care and do not work were excluded from the analysis because only three of the 118 informants who did not work said they had left work because of their care situation. A majority (85) reported they had left work because of their own bad health (not caused by the care situation); other reasons were that they were not satisfied with their work, had lost their job or had taken early retirement.

The parents were predominantly female (84%). Only very few men (5.2%) worked less than a standard workweek, 37.5 h; a third (34.9%) of the women worked less than a standard workweek. More than half among the men (60.4%) reported working more than a standard workweek.

A bit more than one in ten mothers (13%) and fathers (11%) lived in a nursing home. Approximately, four in ten lived in a private home and received home care services (41% of the mothers, 36% of the fathers).

### Measurements

#### Absence from work

Absence from work due to caring for parents was estimated as a latent variable, with three indicators: number of days taken off work to assist parents, irregular work attendance because of assisting a parent and reducing the hours of work to be able to assist a parent. The number of days taken off to assist a parent was measured with an item asking for the number of days, giving responses from 0 to 52, with a very skewed distribution. We recoded answers above five into six (if the original answer was between six and ten, eight respondents) and seven (if the original answer was between 11 and 52, six respondents). Irregular work attendance was assessed with seven items. Three items referred to ‘having been prevented from [participating at meetings or courses/going on business trips/participating in social arrangements related to work]’, one referred to arriving late or having to leave early, one to taking extended breaks, one to avoid overtime or extra work and one to having to change the work schedule in order to assist a parent. All these items were assessed as dichotomous variables and we developed an index variable by summarising the number of ‘yes’ answers. The third indicator, reducing the hours of work (to be able to assist a parent), was a dichotomous indicator assessing whether the respondent had done at least one of the following: taken sick leave to assist a parent over the previous year (2.3% of the sample), reduced the number of work hours over the previous year to assist a parent (1.5%) or taken out leave over the previous year to assist a parent (4.2%).

#### Parent’s health status

We assessed reduced health as a functional impairment and a need for care. We used a measure of ‘Activities of daily living’ (ADL, Katz and Akpom 1976), assessing five classic ADL functions, each with a single item: getting in and out of bed; getting dressed and undressed; grooming; going to the toilet; eating unassisted. Each of these functions was assessed with three-point scales: normally no need for assistance (coded 0); sometimes needs assistance (1) and normally needs assistance (2). We developed an ADL scale by summarising the total number of scores. In addition, respondents indicated whether their parents suffered from dementia (‘No’; ‘Yes, but not severely’; ‘Severely’). The latter two responses were coded as 1 and 2, respectively, and added to the composite variable measuring ADL, resulting in a ADL scale from 0 to 12 (*n* = 485, respondents with missing data not included).

In addition, we used the Instrumental Activities of Daily Living, IADL (Romøren 2004), to index the ability to perform household chores like laundry, cooking, dishwashing. We assessed IADL functions with five items: cleaning, washing clothes or other housework; daily purchases; repairs, gardening, moving snow during the winter and other maintenance work; visits in walking distance from home; taking medicine. Each of these functions was assessed with three-point scales with scores from 0 to 2: normally no need for assistance; sometimes needs assistance; normally needs assistance. The IADL scale was a sum of the scores, resulting in a scale from 0 to 10 (*n* = 464).

#### Public services provided

In addition to assessing the health status of the parent and work absence to assist a parent, we asked whether the parent was living in a nursing home or whether s/he received municipal home care services (during the last 12 months). Both these types of health services were considered as dichotomous variables and entered as a predictor of respondents’ absence from work, as well as a moderator of how the parent’s health situation affected respondents’ absence from work. Dichotomous variables were coded 0 and 1; interaction variables were computed by multiplying the dichotomous variable with mean-centred ADL/IADL scores. Table 1 gives an overview of measured variables, with descriptive statistics and bivariate correlations.

**Table 1:** Descriptive statistics and bivariate correlations

		M	S.D.	Min	Max	Correlations					
						1	2	3	4	5	6
1	Days taken off	0.53	1.47	0	7	1.00					
2	Irregular work attendance	0.53	1.05	0	6	0.35*	1.00				
3	Working hours reduced	0.07	0.26	0	1	0.36*	0.22*	1.00			
4	ADL	1.49	3.11	0	12	0.10*	0.24*	0.03	1.00		
5	IADL	4.24	3.28	0	10	0.16*	0.34*	0.12*	0.64*	1.00	
		Prct.									
6	Nursing home	12.9	na	0	1	−0.01	−0.01	−0.04	0.46*	0.33*	1.00
7	Home-based service	40.2	na	0	1	0.16*	0.22*	0.10*	0.41*	0.59*	0.06

The table uses spearman correlations (with Bonferroni-adjusted significance level) because nursing home and home-based service were dichotomous variables, and also because other variables were skewed. Days taken off is the number of days taken off due to assisting a parent (with 8 answers indicating 6 to 10 days recoded as 6, and 6 answers indicating 11 to 52 days recoded as 7). Irregular work attendance refers to irregular attendance at work-related meetings or courses due to assisting a parent. Working hours reduced is a dichotomous variable combining several indicators. ADL is an index of the parent’s ability to perform ‘activities of daily living’, IADL is an index of the parent’s ability to perform ‘instrumental activities of daily living’, see the Methods section for details. Nursing home refers to the parent living in a nursing home (1 = yes), home-based service refers to the parent receiving home-based care services

* *p* < 0.05

#### Additional items

The questionnaire also included items on whether respondents helped their parent in practical ways (such as cleaning, repairs, purchases, transportation), gave their parent financial assistance, helped with personal hygiene or provided emotional support. All these items provided possible responses ‘Yes, frequently’, ‘Yes, once in a while’ and ‘No’. The questionnaire also included an item asking whether combining care and work was difficult or not (with possible answers ‘Easy’ and ‘Difficult’ for those who provided help). We report simple descriptives for these items.

Some analyses also included an item asking respondents for the number of vacation days used to help parents, used as an auxiliary variable to improve analyses that used multiple imputation but the auxiliary variable was part of the model. We also conducted analyses that considered age, education level and income as control variables (i.e. adding such variables as covariates to the model to see if they affected the ability of public services and parent’s health status to predict absence from work).

### Analysis

We used structural equation modelling, SEM (Bollen 1989; Kline 2011), to analyse the data and estimate absence from work as a latent variable. Data included both continuous and categorical dependent variables. We used Mplus 7.3 (L. K. Muthén and Muthén 2012) for all analyses and applied Mplus’ default estimator weighted least squares means and variance adjusted estimator (WLSMV) for models with a mixture of continuous and categorical data. For one model that did not converge with WLSMV estimation, we used Bayesian estimation. This use of Bayesian estimation was simply to avoid non-convergence for one model (see Muthén 2010); the analysis was thus comparable to a conventional analysis (we did not use informative priors). All analyses incorporated cases with missing data and we applied full information estimations to include cases with partly missing data (Enders 2010). Since Mplus omits cases with missing data on predictors, we also added analyses that applied multiple imputation to generate data for ADL and IADL when scores were missing. We estimated the original models with Mplus but added the command to impute data for the variables ADL or IADL, respectively, using as auxiliary variable the number of vacation days spent to help parents. We compared the results obtained when using multiple imputation with the original analyses.

We used fit indices commonly applied in SEM, using the Chi-square as well as indices of approximate fit. We used a cut-off of 0.95 for the comparative fit index, CFI (Mueller and Hancock 2010). We aimed at models with the root mean square error of approximation (RMSEA) lower than 0.05 but also considered RMSEA up to 0.08 as acceptable; a RMSEA close to 0.08 is highlighted in a table as indicating moderate fit.

## Results

### Helping the parents

Most of the respondents (80%) had provided assistance to their parent, mostly practical (e.g. repairs, purchases, transportation). Relatively few (16%) had given some form of nursing assistance. More than half (58%) of those who reported helping their parent during the last year maintained that it was difficult for them to combine care and work; results were relatively similar for women (56%) and for men (61%).

There was no statistically significant difference between women and men in taking days off work to help their parent (*p* = 0.33) or reducing hours of work (*p* = 0.09). However, significantly (*p* = 0.02) more men (*M* = 0.65, scale from 0 to 6) than women (*M* = 0.43) reported irregular work participation (not attending meetings, courses, etc.). This particular result is probably explained by many of the women already working part time (34.9% reported working less than 37.5 h a week).

Below we show results of SEM analyses, regressing absence from work on ADL, IADL and public services. Separate analyses increasing the sample size by means of multiple imputation for ADL and IADL (and their interaction effects with nursing home or home-based care), which increased sample size to 519 (or 504 in analyses of home-based care), gave very similar results to analyses without multiple imputation. We report the results from analyses without multiple imputation.

### Public health services to the parents affect respondents’ absence from work

We first tested overall results and combined women and men into the same analysis, thereby increasing sample size and statistical power. Table 2 shows results from five different models (five analyses), with parent’s ADL level and public services as predictors of absence from work (due to assisting a parent). *Model 1* estimated the effect of ADL without covariates, showing no significant effect.

**Table 2:** Absence from work dependent on parents’ ability to perform activities of daily life (ADL) and public services provided to the parent

	Model 1	Model 2a	Model 2b	Model 3a	Model 3b
Factor analysis of					
Absence from work					
Days taken off	1.000f	1.000f	1.000f	1.000f	1.000f
Irregular work attendance	0.644***	0.826***	0.881***	0.746***	0.712***
Working hours reduced	0.564***	0.655***	0.644***	0.595***	0.582***
Predictors					
ADL	0.029	0.066**	0.125***	0.027	0.036
Nursing home		−0.529**	0.135		
Home-based service				0.494***	0.501***
Interaction effect			−0.153**		−0.018
R^2^ Absence from work	.010	.030	.070	.096	.095
Model fit					
Chi-square	1.088	6.900	6.487	3.456	3.566
*df*	2	4	6	4	6
*p*	0.581	0.141	0.371	0.485	0.735
RMSEA	0.000	0.039	0.013	0.000	0.000
CFI	1.000	0.971	0.996	1.000	1.000

Coefficients shown for the factor analysis of absence from work are (unstandardized) factor loadings. *df* degrees of freedom, *RMSEA* root mean square error of approximation, *CFI* comparative fit index, *SRMR* standardised root mean square residual. *N* = 475 (*N* = 466 for home-based services). f means fixed prior to estimation. Separate analyses using multiple imputation to increase sample size provided very similar results

*** *p* < 0.001, ** *p* < 0.01; two-tailed significance tests

*Model 2a* added a second predictor – whether the parent was living in a nursing home or not. By entering nursing home as a covariate, the analysis was able to identify a significant association between parent’s ADL and absence from work to assist the parent (*b* = 0.066, *p* < 0.01), while simultaneously showing a negative association between nursing home for the parent and respondents’ absence from work (*b* = −0.529, *p* < 0.01).

*Model 2b* further added the interaction effect between ADL and nursing home, suggesting an interaction effect (*b* = −0.153, *p* < 0.01). Notably however, having the parent in a nursing home did not reduce the association between parent’s ADL and absence from work. Explained variance for absence from work increased notably (from 3 to 7%), suggesting that adding the interaction effect improved the model.

Table 2 also shows tests of home care services as a covariate (Models 3a and 3b). The analysis indicated no moderating effect by home care services. Instead, home care services to the parents were estimated to have a *positive* association with respondents’ absence from work. This particular result probably reflected that home care services were given to older people in need of practical assistance and that assistance by home care services was not sufficient to substitute assistance by the adult children. We did not have a measurement of the quantity or quality of home care services.

Table 3 shows results from analyses with parents’ IADL as a predictor. R^2^ was higher in these models than in the previous analyses with ADL, as respondents primarily provided practical assistance to their parents. Reduced IADL among parents predicted increased absence from work (*b* = 0.099, *p* < 0.001); adding nursing home as a covariate did not reduce this effect (Model 2a in Table 3). A model estimating a potential moderating effect by nursing home (Model 2b) did not converge correctly unless we used Bayesian estimation.^1^ The model showed only an effect for reduced IADL functions (point estimate = 0.112 [C.I. = 0.075 to 0.153]), not for nursing home or the interaction variable for the two predictors.

**Table 3:** Absence from work dependent on parents’ ability to perform instrumental activities of daily life (IADL) and public services provided to the parent

	Model 1	Model 2a	Model 3a	Model 3b
Factor analysis of				
Absence from work				
Days taken off	1.000f	1.000f	1.000f	1.000f
Irregular work attendance	0.937***	0.939***	0.961***	0.825***
Working hours reduced	0.670***	0.686***	0.636***	0.599***
Predictors				
IADL	0.099***	0.110***	0.098***	0.108***
Nursing home		−0.374*		
Home-based service			0.103	0.137
Interaction effect				−0.006
R^2^ Absence from work	.171	.186	.191	.195
Model fit				
Chi-square	6.661	6.625	7.949	6.186
*df*	2	4	4	6
*p*	0.036	0.157	0.094	0.403
RMSEA	0.072	0.038	0.047	0.008
CFI	0.965	0.980	0.966	0.998

Coefficients shown for the factor analysis of absence from work are (unstandardized) factor loadings. *df* degrees of freedom, *RMSEA* root mean square error of approximation, *CFI* comparative fit index, *SRMR* standardised root mean square residual. *N* = 454 (N = 452 for home-based services). Model 1 had moderate fit, with a significant Chi-square and RMSEA = 0.07. f means fixed prior to estimation. Model 2b (testing moderation effect by nursing home) did not converge with maximum likelihood estimation. See the main text for results obtained with Bayesian estimation. Separate analyses using multiple imputation to increase sample size provided very similar results

*** *p* < 0.001, ** *p* < 0.01, * *p* < 0.05, ^ *p* < 0.10; two-tailed significance tests

Table 3 also shows the results of analyses using home care services as a covariate (Model 3a) or as a moderator (Model 3b). Having home care services (a dichotomous variable) correlated at 0.64 with reduced IADL, consistent with an assumption that lower IADL functions are frequently followed up by home care services. However, similar to the previous analysis with ADL functions, the analysis reported in Table 3 failed to indicate that home care service to the parent reduced absence from work.

### Tests of robustness of findings

We conducted several additional tests to investigate the robustness of the findings in Tables 2 and 3 (apart from the previously mentioned used of multiple imputation, inspecting findings with increased sample sizes). Adding covariates such as age, education or income had minimal effect on regression weights in models shown in Tables 2 and 3. Age did not have any association with absence from work, while adding education level or income as a covariate had negligible effects on regression weights for predictors in Tables 2 and 3. Moreover, the results were very similar when we reduced the analysis to include only respondents up to 60 years (omitting respondents who might have considered retirement confirmed the findings reported).

### Comparison of women and men

Table 4 shows the results of analyses that distinguished between women and men as caregivers. Regression weights for ADL, IADL and nursing home were higher for women than for men. These differences, however, were not statistically significant in the analysis involving ADL functions as a covariate to nursing home (*p* = 0.68 for fixing ADL effects to be equal; *p* = 0.19 for fixing effects of nursing home to be equal; *p* = 0.41 for fixing both effects to be equal across gender). In contrast, the analysis using IADL as a covariate showed a significant gender difference for having the parent in a nursing home (*p* = 0.02). For women, a substantial association between nursing home for the parent and non-attendance at work was uncovered (*b* = −0.86, *p* < 0.01) when IADL was used as a covariate. No effect was uncovered for men when IADL was used as a covariate (*b* = 0.00, *p* = 0.996). Consequently, the analysis indicated that women who had a parent with low IADL functions reduced their attendance at work, but to a lesser extent if the parents lived in a nursing home.^2^

**Table 4:** Gender differences in effects of ADL/IADL and nursing home

	ADL		IADL	
	Women	Men	Women	Men
Predictors				
ADL/IADL	0.077*	0.059^	0.150***	0.084**
Nursing home	−0.710*	−0.189	−0.864**	−0.001
Factor analysis of				
Absence from work				
Days taken off	1.000f	1.000f	1.000f	1.000f
Irregular work attendance	0.810***	0.810***	0.821***	0.821***
Working hours reduced	0.719***	0.719***	0.662***	0.662***
Predictors				
ADL/IADL	0.077*	0.059^	0.150***	0.084**
Nursing home	−0.710*	−0.189	−0.864**	−0.001
Intercepts				
Days off	0.435	1.227	−0.032	0.252
Irregular attendance	0.408**	1.093	−0.019	−0.008
Absence	0.000f	−0.622	0.000f	0.259
Residual variances				
Days off	1.133***	1.870***	1.116***	1.962***
Irregular attendance	0.476***	0.803***	0.455***	0.895***
Absence	0.605***	0.623**	0.559***	0.560**
Model fit				
Chi-square total	12.684		9.649	
Chi-square group-specific	11.497	1.187	3.020	6.629
*df*	9		9	
*p*	0.177		0.380	
RMSEA	0.042		0.018	
CFI	0.971		0.996	

*df* degrees of freedom, *RMSEA* root mean square error of approximation, *CFI* comparative fit index, *SRMR* standardised root mean square residual. *N* = 475 (*N* = 466 for home-based services). Metric invariance, that is, invariant factor loadings across gender, was supported (*p* = 0.24), supporting a group-based analysis fixing factor loadings to be equal across the genders. Estimations fixed the threshold for the dichotomous indicator work reduction to be invariant across groups. f means fixed prior to estimation. Coefficients shown for the factor analysis of absence from work are (unstandardized) factor loadings

*** *p* < 0.001, ** *p* < 0.01, * *p* < 0.05, ^ *p* < 0.10; two-tailed significance tests

Separate tests of home care service distinguishing between the genders found no significant difference between women and men in associations between home care service for their parents and absence from work.

## Discussion

This research set out to investigate how public care services for older people affect their adult children’s work attendance. Overall, the data indicated that institutional care offered to older people can have a positive effect on their children’s work attendance. In particular, having a parent in a nursing home was associated with higher work attendance among their children, both as a direct effect and by moderating the link between the parents’ reduced health as a predictor and work attendance as a dependent variable. We also found a gender effect in that the effect of nursing homes for parents with low IADL functioning was stronger for the daughters’ than for the sons’ work attendance.

However, the results differed for home care services. Having a parent who received home care was associated with reduced work attendance. This finding might seem paradoxical, but it probably reflects an important aspect of public care provided to older people. Home care services are given to older people with a greater need for help, but these services are probably insufficient. Thus, since the provision of (public) home care services is a consequence of higher care needs among the parents, we find a seemingly negative effect of home care services on children’s work participation because the children have to step in and compensate for a gap between the need for care and the assistance provided by public non-institutionalised services.

### Policy Implications

Increased life expectancy and female labour market participation have resulted in an increasing number of 50- and 60-year-olds combining work with care for their parents. Knowledge is scant on policies that might facilitate work participation and occupational efforts among caring senior employees (Gautun and Hagen 2010; Kröger and Yeandle 2013).

Many Western societies aim at increased labour market participation among senior employees (Gautun and Hagen 2010; Naldini et al. 2014). Little attention has been given to the potential importance of public care services for the parents of senior employees for reducing part-time work, early retirement or absence from work. Our study indicates that employees have less absence from work when their parent in need of care lives in a nursing home; this seems particularly true for daughters of older people. We did not find a similar effect with home care services. Together, these results suggest that the public care given to older people needs to reach a certain level to prevent their daughters from reducing work attendance or even withdrawing from labour market participation.

These findings are consequential for the ongoing debate regarding institutionalised care. Since the early 1990s there has been a trend in many Western countries towards de-institutionalisation of health and care services. Figures from Statistics Norway show that in 1991, 50% of the population aged 90 or more were living in institutions that provided long-term care. By 2011 this proportion had been reduced to 30% (Ram 2013). The implied reduced care has not been compensated for by improvements in home care services, however. On the contrary, adjusted for the increased number of older people, home care services for the oldest population have been reduced (Gautun and Grødem 2015). Without substantial improvement in home care services to older people, de-institutionalisation is likely to result in older people not receiving the care they need. Many employees (in particular women) are likely to feel they have to compensate for the inadequate care of their parents, with or without an explicit request from the parents themselves.

Home care services to older people probably need to be extended if they are to be a realistic alternative to institutionalised care. Moreover, care for older people seems to require modernisation. Employees with parents in need of care report that a more flexible health and care system with extended opening hours and the possibility of home visits by general practitioners would ease their efforts to accompany parents to a hospital or doctor after work (Gautun and Hagen 2010). Modernisation could also be in the form of new services for older people. For instance, some municipalities in Norway have established day centres for certain groups of older people, which make it easier for their adult children to focus on work tasks while at work. Currently, the number of day centres for older people is limited, but an extension would contribute to daughters and sons having less absence from work.

### Future Research

A strength of the present research is that it is innovative in investigating how public health services to older people affect their children’s work attendance, and how employees combine work with care for elderly parents. However, as an innovative study, it had limitations which should be dealt with in future research. All measured variables relied on reports from children of older people, and data are cross-sectional. Improved measurements of variables, including more objective assessments of work attendance, will help. The same is true in relation to using longitudinal instead of cross-sectional data. We also assume that improving measurements will increase the explained variance in the dependent variable absence from work (7% in this study).

Since nursing homes and home-based care are funded and allocated by the public sector in Norway, the causality assumed in the present research seems plausible. Thus, the present research does not suffer from the ambiguous causalities that characterise research in countries where nursing homes or home-based care are privately funded. There is no reason to assume a reversed causality, where children of older people might use nursing homes (or home-based care) for their parents in order to maintain full employment for themselves (see Bonsang 2009 for a discussion of potential problems related to causality in this research field). Nursing homes and home-based services in Norway are allocated based on the older person’s needs, not on their adult children’s preferences. Nevertheless, future research that uses longitudinal data—testing how changes in public care to older people is associated with developments over time in the children’s work attendance—would improve investigation into causalities.

One limitation, however, is that we were unable to investigate possible variations between different parts of the labour market. Comparative research shows significant variation in provision of family-friendly working arrangements, leading to unequal access to such flexibility across workplaces (Warth 2008). For instance, a British study found that that family-friendly or flexible working arrangements were more common in larger companies, the public sector, recognised unisons and sectors with higly educated employees (Dex and Smith 2002). Norway has advanced public care for older people as well as strong rights for employees. Still, these employee rights and the ability to combine work with care for older parents may vary between different parts of the labour market. Our data did not allow for investigations into potential differences between different parts of the labour market, a task that can be followed up by future research in Norway. Identifying variations between different parts of the labour market will be crucial in formulating labour and welfare policies that are adapted to the needs of different groups of employees.

Public health services can also be assessed in more detail. The present research relied on dichotomous variables assessing the presence of public care services; future research might use quantified measures of home care service and test how the level of home care service for parents is associated with their adult children’s work attendance. In particular, we argue that home-based care given to older people is insufficient to meet their needs, resulting in a statistical association between older people receiving home-based care and reduced work attendance among the children. Future research can test this explanation with more detailed measures of health among older people, the level of home-based care provided and work attendance among their adult children.

## Footnotes

1 We used non-informative priors and 100,000 iterations; the potential scale reduction was consistently <1.01 after 10,500 iterations. The Posterior Predictive P-Value was 0.428.

2 Models adding an interaction effect between ADL/IADL and nursing home could not be estimated without multiple imputation (to increase sample size, using the previously mentioned input for multiple imputation). When using multiple imputation and adding an interaction effect in both models mentioned in Table 4, only the main effect of nursing home was estimated to be statistically significantly different across the genders, now in the analysis using ADL as a covariate (*p* = .044).
